# Development of an improved *Pseudoalteromonas haloplanktis *TAC125 strain for recombinant protein secretion at low temperature

**DOI:** 10.1186/1475-2859-7-2

**Published:** 2008-02-07

**Authors:** Ermenegilda Parrilli, Daniela De Vizio, Claudia Cirulli, Maria Luisa Tutino

**Affiliations:** 1 Dipartimento di Chimica Organica e Biochimica, Università degli studi di Napoli Federico II – Complesso Universitario M.S. Angelo via Cinthia 4, 80126, Napoli Italia; 2 Facoltà di Scienze Biotecnologiche, Università degli studi di Napoli Federico II, Napoli Italia; 3 Dipartimento di Biotecnologie e Bioscienze dell'Università Milano-Bicocca, Piazza della scienza 2, 20126, Milano Italia

## Abstract

**Background:**

In a previous paper, we reported the accomplishment of a cold gene-expression system for the recombinant secretion of heterologous proteins in *Pseudoalteromonas haloplanktis *TAC125. This system makes use of the psychrophilic α-amylase from *P. haloplanktis *TAB23 as secretion carrier, and allows an effective extra-cellular addressing of recombinant proteins. However, *Pseudoalteromonales *are reported to secrete a wide range of extra-cellular proteases. This feature works against the efficiency of the cold-adapted secretion system, because of the proteolytic degradation of recombinant products. The aim of this study is the construction of a *P. haloplanktis *TAC125 mutant strain with reduced extra-cellular proteolytic activity.

**Results:**

*P. haloplanktis *TAC125 culture medium resulted to contain multiple and heterogeneous proteases. Since the annotation of the Antarctic bacterium genome highlighted the presence of only one canonical secretion machinery, namely the Type II secretion pathway (T2SS), we have inactivated this secretion system by a gene insertion strategy. A mutant strain of *P. haloplanktis *TAC125 in which the *gspE *gene was knocked-out, actually displayed a remarkable reduction of the extra-cellular protease secretion. Quite interestingly this strain still retained the ability to secrete the psychrophilic amylase as efficiently as the wild type. Moreover, the decrease in extra-cellular proteolytic activity resulted in a substantial improvement in the stability of the secreted amylase-β-lactamase chimera.

**Conclusion:**

Here we report a cell engineering approach to the construction of a *P. haloplanktis *TAC125 strain with reduced extra-cellular protease activity. The improved strain is able to secrete the psychrophilic α-amylase (the carrier of our recombinant secretion system), while it displays a significant reduction of protease content in the culture medium. These features make the *gspE *mutant an improved host with a remarkable biotechnological potential in recombinant protein secretion at low temperature. Moreover this work demonstrates that *P. haloplanktis *TAC125 is a versatile psychrophilic host for recombinant protein production since it can be easily improved by a directed engineering approach. To the best of our knowledge, this is the first described example of a strain improvement strategy applied to an Antarctic bacterium.

## Background

Protein secretion into the extra-cellular environment is one of most desirable strategy to allow a rapid and not expensive recovery of recombinant proteins. Secretion to the culture medium has several advantages over intracellular recombinant protein production. These advantages include simplified downstream processing, enhanced biological activity, higher product stability and solubility, and N-terminal authenticity of the expressed peptide [1-3]. If the product is secreted to the culture medium, cell disruption is not required for recovery. As bacteria, usually, do not secrete amounts of proteins higher then they have in the intracellular space, recovery of a recombinant gene product can be greatly simplified by a secretion strategy that minimises contamination from host proteins. Additionally, secretion can provide a method to guarantee the N-terminal authenticity of the expressed polypeptide because it often involves the cleavage of a signal sequence [4], thus avoiding the presence of an unwanted initial methionine on a protein that does not normally contain it. This extra methionine can reduce the biological activity and stability of the product [5] or even elicit an immunogenic response in the case of therapeutic proteins.

In a previous paper [6], we reported the realization of a "cold" recombinant secretion system in the Antarctic Gram-negative bacterium *P. haloplanktis *TAC125. This system efficiently conjugates the obvious advantages of extra-cellular protein targeting with the positive effect of low temperature on the recombinant product solubility. Indeed, low expression temperature can facilitate the correct folding of "difficult" products [7,8] and the use of *P. haloplanktis *TAC125 as expression system [9] allowed the efficient production of some "intractable" proteins in soluble and active form at temperature as low as 4°C [10-12].

The cold-adapted secretion system [6] makes use of the psychrophilic α-amylase from *P. haloplanktis *TAB23 [13,14] as secretion carrier. Three chimerical proteins, made of the psychrophilic α-amylase fused to an intra-cellular protein, were translocated in the extra-cellular medium with a secretion yield always higher than 80%. The system also allowed the correct disulphide bond formation of chimera components, secreting a fully active passenger [6]. However, our previous results addressed to a potential limit of this newly set up technology: host extra-cellular medium may contain proteolytic activities which can affect the quality of heterologous products [6]. This feature could hamper the applicability of the cold-adapted secretion system, due to the likely recombinant product degradation.

To overcome this host limit, two possible approaches can be pursued thanks to the availability of *P. haloplanktis *TAC125 genome sequencing and annotation [15]: i) the gene disruption of each *P. haloplanktis *TAC125 gene encoding extra-cellular proteases; or ii) the inactivation of the secretion machinery responsible for the proteases extra-cellular targeting.

The *in silico *analysis of *P. haloplanktis *TAC125 genome demonstrated that the bacterium possesses only one canonical secretion system, a putative Type II secretion machinery (T2SS) also called General Secretory Pathway (GSP), homologous to GSPs already described in many other Gram-negative bacteria [16]. Since experimental evidences suggested that in *P. haloplanktis *TAC125 the secretion of the cold α-amylase depends on a still uncharacterized pathway (unpublished results from this laboratory), the inactivation of the T2SS machinery seemed a reasonable strategy to develop a *P. haloplanktis *TAC125 mutant strain with reduced extra-cellular proteolytic activity.

In this paper we report the set-up of an integrative plasmid and its use for the construction of a *P. haloplanktis *TAC125 strain in which the *gspE *gene [17,18] was knocked-out. This mutation resulted in the inactivation of the psychrophilic T2SS system. The *P. haloplanktis *TAC125 *gspE *mutant displayed a remarkable reduction of the extra-cellular protease secretion, still maintaining its ability to secrete the psychrophilic amylase (the secretion carrier of our recombinant system) as efficiently as the wild type. These features make the *P. haloplanktis *TAC125 *gspE *mutant strain an improved host with a remarkable biotechnological potential in recombinant protein secretion at low temperature.

## Results

### *Pseudoalteromonas haloplanktis *TAC125 growth medium contains several secreted proteases

**Figure 1:**
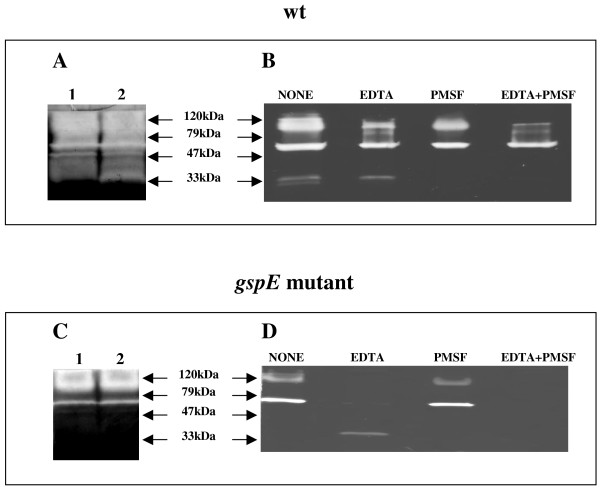
**In gel analysis of extra-cellular proteolytic activities from culture supernatants of *P. haloplanktis *TAC125 wild type and *gspE *mutant strain**. Panel A: Zymography of *P. haloplanktis *TAC125 wild type culture supernatants collected at early (24 h) (lane 1), and middle (32 h) (lane 2) exponential phase. In this experiment the zymographic developing time was 18 h, a condition that assures the detection of all proteases contained in the sample. Panel B: Protease zymography of a *P. haloplanktis *TAC125 wild type culture supernatant, collected at 24 h, untreated (NONE) and treated with protease inhibitors (10 mM EDTA, 10 mM PMSF, and the combination of the two inhibitors both at 10 mM final concentration). In this experiment a zymographic developing time of 12 h was chosen, this condition allows a clearer visualization and comparison of the proteases contained in the different samples. Panel C: Protease zymography of *P. haloplanktis *TAC125 *gspE *mutant culture supernatants collected at early (24 h) (lane 1), and middle (32 h) (lane 2) exponential phase, the zymographic developing time was 18 h. Panel D: Protease zymography of a *P. haloplanktis *TAC125 *gspE *mutant culture supernatant, collected at 24 h, untreated (NONE) and treated with protease inhibitors, the zymographic developing time was 12 h).

Wild type *P. haloplanktis *TAC125 cells were grown in TYP medium at 4°C and culture medium samples were withdrawn at different growth phases (at 24, and 32 hours of incubation corresponding to early and medium exponential phase). Concentrated culture supernatants were analyzed for proteolytic activities using Gelatine-SDS-PAGE (10% acrylamide, w/v) as described in Materials and Methods. As shown in Figure 1A, the wild type *P. haloplanktis *TAC125 culture supernatants contain a wide range of proteolytic activities, which display an apparent molecular weight ranging between 120 and 33 kDa.

A protease inhibition assay was performed by treating the 24 hours extra-cellular protein sample with 10 mM PMSF (a serine protease inhibitor) for 20 hours at 15°C, 10 mM EDTA (a metalloprotease inhibitor) for 20 hours at 15°C, and with the combination of the two inhibitors. The differentially treated samples were then subjected to gelatine zymography, and the results are shown in Figure 1B. A comparative evaluation of the results demonstrates that the wild type *P. haloplanktis *TAC125 culture supernatant contains extra-cellular proteases inhibited either by PMSF or by EDTA, and proteolytic activities which appear to be resistant to both the inhibitors (Figure 1B lane EDTA+PMSF).

### *Pseudoalteromonas haloplanktis *TAC125 genome encodes a functional Type II protein secretion system

Annotation of *P. haloplanktis *TAC125 genome sequence [15] revealed that the psychrophilic bacterium possesses only a canonical extra-cellular protein secretion pathway, i.e. the Type II secretion system (T2SS). The psychrophilic T2SS is located on the larger *P. haloplanktis *TAC125 chromosome and it is made up of twelve genes, from the gene PSHAa0231 to PSHAa0242. The *gsp *gene cluster is likely arranged in several independent transcriptional units, as previously observed in other Gram-negative bacteria [19,20]. Gene expression of *P. haloplanktis *TAC125 T2SS encoding cluster was evaluated by Reverse Transcriptase (RT)-PCR. Total RNA was extracted from cell samples collected at different growth phases, and was used to assess the transcription of *gspE*, *gspC*, and *gspN*. All the tested *gsp *genes resulted to be constitutively expressed in *P. haloplanktis *TAC125 cells (data not shown).

### Construction and genetic characterization of *Pseudoalteromonas haloplanktis *TAC125 *gspE *mutant strain

**Figure 2:**
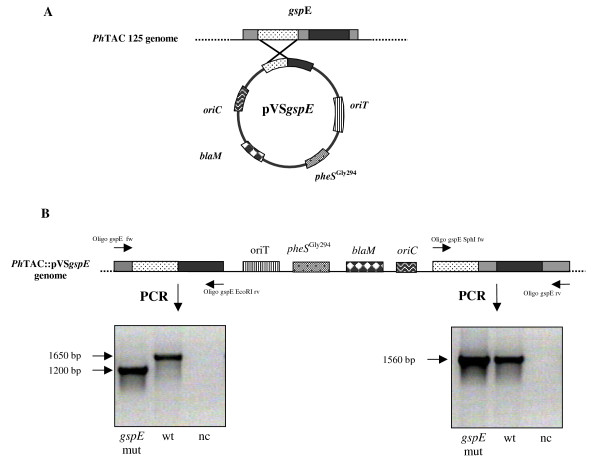
**Schematic representation of pVS suicide vector (panel A) and genetic organization of *P. haloplanktis *TAC125 *gspE *mutant (panel B)**. See text for details.

Functional inactivation of *P. haloplanktis *TAC125 T2SS system was achieved by insertional mutagenesis of *gspE *gene. This target was selected because it encodes an inner membrane-associated ATP-synthase, which has previously been reported to be essential for the T2SS functioning in other bacteria [21]. Insertional mutagenesis was obtained by using a suicide vector (pVS), suitably constructed for *P. haloplanktis *TAC125. As shown in Figure 2A, the pVS vector is characterized by the presence of: i) the pJB3-derived *oriT *[22], a DNA fragment responsible for the initiation of the conjugative transfer between an *Escherichia coli *S17-1 λ*pir *strain (donor) and the psychrophilic cells (acceptor); ii) the *E. coli blaM *gene, encoding a mesophilic β-lactamase which is used as selection gene to isolate the first site-specific integration event; iii) *pheS*^Gly294^, which encodes a mutated version of the *E. coli α *subunit of Phe-tRNA synthase [23], which renders bacteria sensitive to *p-*chlorophenylalanine. This phenylalanine analogue can be used as counterselective agent for the isolation of those strains in which a second recombination event occurred.

Two *gspE *gene fragments were amplified by PCR using specific oligonucleotides as primers. They correspond to two internal gene fragments and they are not adjacent (Figure 2A). The fragments were suitably digested and cloned into the pVS vector. The resulting vector (pVS*gspE*) was mobilized into *P. haloplanktis *TAC125, and clones in which a single recombination event occurred were selected on carbenicellin containing solid medium. Genomic DNA from some carbenicellin-resistant clones was extracted and subjected to different PCR analyses, to characterize the occurred genomic insertion. A positive clone was selected for further characterization, and its genome organization is reported in Figure 2B. *P. haloplanktis *TAC125::VS*gspE *mutant (hereafter called *P. haloplanktis *TAC125 *gspE *mutant) resulted to contain two copies of *gspE *gene, both carrying a specific deletion that was checked by sequencing the specific PCR products shown in Figure 2B. The first copy of *gspE *gene differs from the wild type one in lacking i) the region (450 bp long) between the two amplified fragments and ii) the 3' encoding region (Figure 2B). A transcriptional analysis demonstrated that this *gspE *copy is transcribed (data not shown), but due to the large deletion, the resulting gene contains a translation frame shift leading to the production of an abnormal protein. The other *gspE *gene copy (Figure 2B) lacks of its promoter region and of the 5' encoding portion, and therefore it resulted to be not transcribed (data not shown).

*P. haloplanktis *TAC125::VS*gspE *mutant was subjected to *p-*chlorophenylalanine treatment to select a clean deletion mutant. Although the counter-selection was carried out in several experimental conditions, all the selected clones resulted to be meroploid strains, containing both inserted and wild type alleles (data not shown).

### *Pseudoalteromonas haloplanktis *TAC125 *gspE *mutant strain displays a reduced extra-cellular protease activity

**Figure 3:**
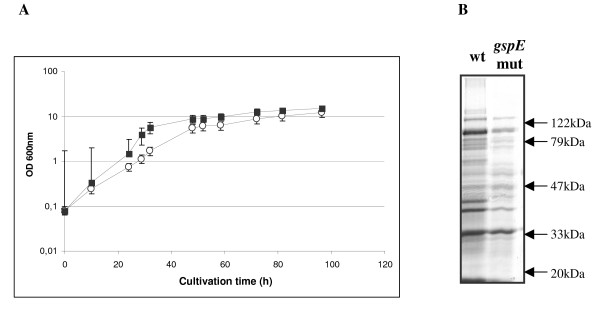
**Comparison of *P. haloplanktis *TAC125 wild type and *gspE *mutant growth kinetics and extra-cellular protein contents**. Panel A: growth kinetics of *P. haloplanktis *TAC125 wild type (open circle) and *gspE *mutant (solid square) in TYP medium at 4°C. Panel B: 12% SDS-PAGE analysis of tenfold concentrated culture supernatants of *P. haloplanktis *TAC125 wild type and *gspE *mutant cells grown for 32 hours.

Growth behaviour of *P. haloplanktis *TAC125 *gspE *mutant strain in standard conditions was determined, and compared with the wild type one. As shown in Figure 3A, the *gspE *mutant strain grows faster and makes a higher cellular biomass than the wild type strain. Concentrated culture supernatants of *P. haloplanktis *TAC125 wild type and *gspE *mutant were analyzed by SDS-PAGE and results are shown in Figure 3B. The *gspE *mutant culture supernatant contains a reduced number of proteins compared to the wild type.

Extra-cellular protease secretion in *P. haloplanktis *TAC125 *gspE *mutant strain was investigated by gelatine zymography of concentrated culture supernatants. As shown in Figure 1C, the samples contain a notably reduced number of proteolytic activities as compared to the protease content of wild type samples (Figure 1A) at the corresponding growth phases. Interestingly, the combined EDTA/PMSF treatment resulted in the almost complete inhibition of the extra-cellular proteases secreted by *gspE *mutant (Figure 1D).

### *Pseudoalteromonas haloplanktis *TAC125 *gspE *mutant strain is an improved host for the recombinant protein secretion at low temperature

**Figure 4:**
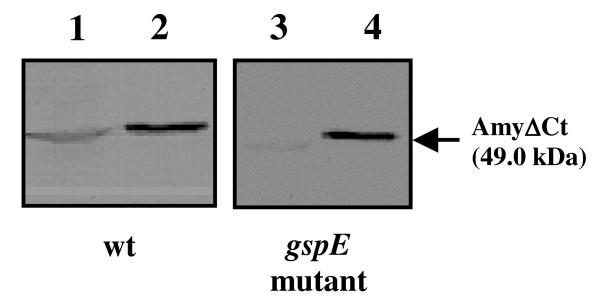
**Psychrophilic α-amylase secretion in *P. haloplanktis *TAC125 *gspE *mutant strain**. Western blotting analysis of extra-cellular media (lane 4) and corresponding cellular extract (lane 3) of *P. haloplanktis *TAC125 *gspE *mutant transformed with pFC*amy*ΔCt plasmid. The western blotting analysis of extra-cellular media (lane 2) and corresponding cellular extract (lane 1) of recombinant *P. haloplanktis *TAC125-(pFC*amy*ΔCt) recombinant cells is shown as a control.

Secretion of psychrophilic α-amylase in *P. haloplanktis *TAC125 *gspE *mutant cells was studied. The mutant strain was transformed with pFC*amy*ΔCt, a psychrophilic vector previously constructed for the recombinant secretion of α-amylase in the Antarctic bacterium [6]. The recombinant mutant strain was grown at 4°C till medium exponential phase and the α-amylase secretion was evaluated by Western blotting analysis of cellular (Figure 4, lanes 3) and extra-cellular (Figure 4, lanes 4) protein samples. The results demonstrated that the *gspE *mutation does not affect secretion of the psychrophilic enzyme.

**Figure 5:**
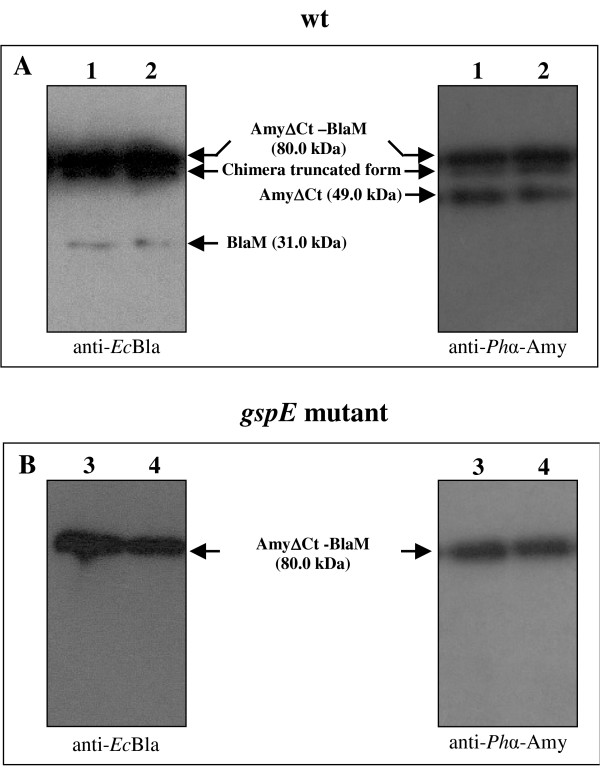
**AmyΔCt-BlaM chimera secretion in *P. haloplanktis *TAC125 wild type and *gspE mutant***. Panel A: Western blotting analyses of extra-cellular media of *P. haloplanktis *TAC125(pFC*amy*ΔCt-*blaM*) recombinant cells. Samples were collected during the early (24 h) (lane 1), and middle (32 h) (lane 2) exponential phase. Immunodetection was performed by chemioluminescence after probing proteins with anti-α-amylase (anti-*Ph*α-Amy) and anti-β-lactamase (anti-*Ec*Bla) polyclonal antisera. Panel B: Western blotting analyses of extra-cellular media of *P. haloplanktis *TAC125 *gspE *mutant-(pFC*amy*Δ*Ct*-*blaM*) recombinant cells. Samples were collected during the early (24 h) (lane 3), and middle (32 h) (lane 4) exponential phase. The immunodetections were performed by using anti-α-amylase (anti-*Ph*α-Amy) and anti-β-lactamase (anti-*Ec*Bla) polyclonal antisera.

The secretion of the chimerical protein AmyΔCt-BlaM, made up of the psychrophilic amylase fused to the mature β-lactamase [6], in *P. haloplanktis *TAC125 *gspE *mutant cells was investigated. *P. haloplanktis *TAC125 *gspE *mutant-(pFC*amy*Δ*Ct-blaM*) recombinant cells were grown in TYP medium at 4°C and samples were collected at different growth phases (early, and medium exponential phase). Culture supernatants were analysed by Western blotting analysis using anti-β-lactamase (anti-*Ec*Bla) and anti-α-amylase (anti*Ph*α-Amy) polyclonal antisera, respectively. As shown in Figure 5B lanes 3 and 4, both antisera detected a single product, with an apparent molecular weight of 80 kDa, corresponding to the AmyΔCt-BlaM chimerical protein. When a similar analysis was applied to wild type *P. haloplanktis *TAC125-(pFC*amy*Δ*Ct-blaM*) recombinant cells (Figure 5A lane 1 and 2), culture supernatants contained either the chimerical protein or its different proteolysis products, i.e. AmyΔCt, BlaM, and a chimera truncated form due to the action of host-encoded secreted proteases on the passenger protein.

## Discussion

In a previous paper [6], we reported the establishing of a versatile gene-expression system for secretion of heterologous proteins in *P. haloplanktis *TAC125. The system uses the psychrophilic α-amylase from *P. haloplanktis *TAB23 as secretion carrier, and allows an efficient extra-cellular addressing of recombinant proteins. However, we realized that the system efficiency was hampered by the presence of host-encoded extra-cellular proteolytic activities [6]. The presence of extra-cellular proteases represents a severe limit to the use of any genetic system for the recombinant protein secretion, since host-encoded proteases can affect the quality of the heterologous secreted products.

To enhance the recombinant product quality and stability, we focused our attention on the extra-cellular proteases produced by the psychrophilic *P. haloplanktis *TAC125 with the aim of developing a host mutant strain with reduced extra-cellular proteolytic activity.

By in gel activity assay, we demonstrated that several proteases are present in the culture medium of the psychrophilic bacterium (Figure 1A). The zymographies presented in Figure 1 did not allow us to define the exact number of extra-cellular proteases, due to the low resolution of this experimental technique. However, the inhibition assay allowed us to explore at least the diversity in the action mechanisms of the psychrophilic extra-cellular proteases. Indeed, metalloproteases, serine proteases and enzymes belonging to different classes were detected (Figure 1B).

Considering the multiplicity and heterogeneity of proteases detected in *P. haloplanktis *TAC125 culture medium, the systematic disruption of each extra-cellular protease encoding gene appears a time-consuming strategy. Therefore, we decided to inactivate the molecular machinery responsible for the extra-cellular targeting of proteases.

This aim was achieved combining some information deriving from the *in silico *analysis of *P. haloplanktis *TAC125 genome with several experimental evidences. In particular, from the accurate genome annotation, it was known that the psychrophilic bacterium possesses only one canonical secretion machinery (the T2SS pathway), while we have collected evidences indicating that the psychrophilic α-amylase (the secretion carrier of our recombinant system) is likely secreted by another secretion apparatus not yet fully characterized (unpublished results from this laboratory). Thus, the functional inactivation of *P. haloplanktis *TAC125 T2SS seemed a feasible approach to generate a psychrophilic mutant strain possibly secreting a lower protease amount but still able to secrete the recombinant α-amylase and its chimerical derivates.

T2SS (also called General Secretory Pathway) is a multi-component machinery encoded by the *gsp *cluster and promoting secretion of Sec- and Tat-dependent exo-enzymes in a two-step process [16,19,20]. Our results demonstrated that the *P. haloplanktis *TAC125 *gsp *gene cluster is actually transcribed and that the *gsp *genes expression seems to be constitutive over the bacterial growth (data not shown). Therefore it was necessary to use a genetic approach aimed at completely abolishing *gsp*-dependent secretion during all growth phases. *gspE *Gene [17,18] encodes a specialized ATP-synthase whose inactivation resulted in the total loss of T2SS functionality in other Gram-negative bacteria [21,24,25]. Therefore the psychrophilic *gspE *gene was selected as target for inactivation by insertional mutagenesis.

The *P. haloplanktis *TAC125 *gspE *mutant strain was constructed by applying a typical gene targeting strategy, which makes use of a suitably constructed psychrophilic suicide vector (Figure 2A). As described in result section, this mutagenesis strategy allowed the creation of a *P. haloplanktis *TAC125 mutant strain characterized by the absence of GspE function.

A preliminary phenotypic analysis, carried out in standard growth conditions, demonstrated that *P. haloplanktis *TAC125 *gspE *mutant displays specific growth rate and biomass productivity higher than the wild type strain, thus surprisingly the mutation does not affect but improve the bacterium fitness (Figure 3A). This observed *gspE *mutant behaviour could be justified considering that *gsp*-dependent protein secretion is a high costly metabolic process, and its inhibition may represent an advantage to *gspE *mutant strain, at least in the tested growth condition.

The analysis of the extra-cellular protein content demonstrated that *gspE *mutant actually secretes lower amounts of proteins with respect to the wild type strain (Figure 3B). Proteins secreted by the *gspE *mutant are translocated by secretion pathways different from T2SS, such as the specialized machineries for type IV pili and curli components secretion [15] and the secretion apparatus responsible for the recombinant α-amylase secretion (unpublished results from this laboratory).

Zymographic analysis revealed that the *gspE *mutant strain medium contains a notably reduced number of proteolytic activities (Figure 1C) with respect to wild type culture supernatants (Figure 1A). In contrast, knocking out of *gspE *gene did not impair for secretion of the cold-adapted amylase (Figure 4). In fact, *P. haloplanktis *TAC125 wild type and *gspE *mutant strains resulted to be indistinguishable in terms of cold-adapted α-amylase production and secretion yields.

The *gspE *mutant was further tested for its ability to secrete a α-amylase chimerical product, the AmyΔCt-BlaM chimera. When produced by wild type cells, the chimera components (i.e. the psychrophilic α-amylase and the mesophilic β-lactamase) (Figure 5A, lanes 1 and 2) are partially separated due to the sub-stoichiometric proteolytic cleavage of the linker which connects the two proteins (see also [6]). This processing does not affect the catalytic activity of each component of the chimera [6]. The host-encoded secreted proteases are also responsible for further chimera degradation (see "chimera truncated form" in figure 5A, lanes 1 and 2) which accounts for the previously reported and undesired decrease of passenger activity [6]. On the contrary, due to the reduced number of extra-cellular proteases present in culture medium, AmyΔCt-BlaM chimera accumulates as a unique unprocessed form when produced in *P. haloplanktis *TAC125 *gspE *mutant cells (Figure 5B, lanes 3 and 4), thus resulting in an enhancement of chimera quality over the whole production process.

## Conclusion

Combining the experimental evidences we collected on the α-amylase secretion machinery in *P. haloplanktis *TAC125 with the careful *in silico *analysis of its genome, we designed an simple and successful experimental approach for the construction of an improved psychrophilic host for the cold α-amylase-dependent recombinant secretion system. In fact, by a single gene disruption, involving the psychrophilic *gspE *gene, we developed a *P. haloplanktis *TAC125 mutant strain which secretes a significantly reduced extra-cellular protease activity while keeping its ability to secrete the recombinant psychrophilic α-amylase as the wild type strain. The *gspE *mutant strain is also characterized by specific growth rate and biomass productivity higher than wild type strain, making it a truly improved host with a remarkable biotechnological potential in recombinant protein secretion at low temperature. Moreover, this work demonstrates that *P. haloplanktis *TAC125 is a versatile psychrophilic host for recombinant protein production since it that can be easily improved by a directed engineering approach. To the best of our knowledge, this is the first described example of a strain improvement strategy applied to an Antarctic bacterium.

## Methods

### Strains and plasmids

*P. haloplanktis *TAC125 was isolated from Antarctic sea water [15]. *Escherichia coli *DH5α [26] was used as host for the gene cloning. *E. coli *strain S17-1(λ*pir*) was used as donor in interspecific conjugation experiments [27].

### Growth conditions and analytical procedures

*P. haloplanktis *TAC125 was grown in aerobic conditions at 4°C in TYP broth (16 gr/L yeast extract, 16 gr/L bacto tryptone, 10 gr/L marine mix) at pH 7.5, supplemented with ampicillin 200 μg/ml, chloramphenicol 25 μg/ml, or cabenicellin 30 μg/ml, when required. Antarctic bacteria transformation was achieved by intergeneric conjugation as previously reported [9].

*E. coli *cells were routinely grown in Terrific broth [28] at 37°C. When required, antibiotics were added at the following concentrations in liquid cultures: 100 μg/ml of ampicillin, or cloramphenicol at 50 μg/ml final concentration. Genetic manipulations were carried out following standard procedures [28].

*P. haloplanktis *TAC125 DNA genomic purification was performed by ChargeSwitch gDNA Mini Bacteria Kit (Invitrogen).

Protein samples were analyzed by Polyacrylamide Gel Electrophoresis (Sodium Dodecyl Sulphate-PAGE) (12% acrylamide, w/v) according to standard methods [28]. For immunoblotting, the proteins were transferred to a polyvinylidene difluoride membrane (Immobilon PSQ, Millipore). For immunodetection of proteins, *P. haloplanktis *TAB23 anti-α-amylase [29] or anti-β-lactamase antisera were diluted in blocking buffer (phosphate buffer saline; 5% skimmed milk). Peroxidase conjugate anti-rabbit IgG (Sigma-Aldrich, USA) was used as secondary antibody. Proteins were detected by chemiluminescence's (Pierce, USA).

### Construction of suicide insertion vector pVS*gspE*

pVS suicide vector was constructed by the insertion of the pJB3-derived *oriT *[22] and *pheS*^Gly294 ^gene [23] into the pGEM7Z vector. The *oriT*, responsible for the initiation of the conjugative transfer, was amplified on pJB3 vector by using Oligo oriTEcoRIfw and Oligo oriTSacIrv as primers (see Table 1), and was cloned into *EcoRI *and *SacI *sites of pGEM7Z (pGEM7Z-OriT). *pheS*^Gly294 ^gene, which encodes a mutated version of the *E. coli α *subunit of Phe-tRNA synthase, was amplified using pKSS [23] vector as template. The PCR reaction was carried out using the oligonucleotide pair PheSSNfw and PheSXrv, designed to introduce *Nde*I and *Xba*I restriction sites. The amplified DNA fragment was subjected to double *Nde*I/*Xba*I digestion and cloned into pPM13 plasmid [11] corresponding sites generating pPM13-*pheS*^Gly294 ^vector. The DNA fragment, containing P13 promoter and *pheS*^Gly294 ^gene, was recovered from pPM13-*pheS*^Gly294 ^vector by *SmaI/EcoRI *digestion, filled in and cloned into the pGEM7Z-*oriT NaeI *restriction site, resulting in the construction of the pVS vector.

**Table 1:** Plasmids and oligonucleotides used in this work

*Plasmids*		
pFC	Psychrophilic gene-expression vector, containing the T/R box, the promoter and termination region of the *P. haloplanktis *TAC125 *asp*C gene and the chloramphenicol resistance gene	[6]
pFC*amy*ΔCt	pFC containing a truncated version of the *amy *gene devoid of the C-terminal propeptide encoding portion	[6]
pFC*amy*ΔCt-*blaM*	pFC containing the *amy*ΔCt-*blaM *gene encoding the chimerical protein Amy-BlaM	[6]
pVS	pGEM7Z vector containing a conjugation transfer origin (oriT), the counter selectable marker pheS ^Gly294 ^and the ampicillin resistance gene	This work
*Oligonucleotides*		
Oligo oriT*Eco*RIfw	5'-TTGAATTCTCGCACGATATACAGG-3'	
Oligo oriT*Sac*Irv	5'-AAGAGCTCTTGAAGACGAAAGGG-3'	
Oligo gspE*Sph*Ifw	5'-TTGCATGCATGCGCATCATCCGG-3'	
Oligo gspE*Sac*Irv	5'-AAGAGCTCCAATATCGAGCTTAGCC-3'	
Oligo gspE*Sac*Ifw	5'-TTGAGCTCCTAAAGTAGGTATGACC-3'	
Oligo gspE*Eco*RIrv	5'-AAGAATTCGTACACGGGCTACAGCC-3'	
Oligo gspESrv	5'-AAGAGCTCCTTCACTGAGCATCG-3'	
Oligo gspEfw	5'-GCAATTTAAGCAGCGCGAAGATG-3'	
Oligo gspErv	5'-ATCTAGGGCACGGTATTCAAATGC-3'	
PheSSNfw	5'-TTGTCGACATATGTCACATCTCGCAGAAC-3'	
PheSXrv	5'-CCTCTAGAGAATTTCATAATCTATTCCTGCC-3'	

Two DNA fragments of *P. haloplanktis *TAC125 *gspE *gene were amplified by PCR using bacterial genomic DNA as template. Two primer pairs were designed to amplify a 567 bp region at the 5' end (Oligo gspE*Sph*Ifw, Oligo gspE*Sac*Irv) and a 621 bp region at the 3' (Oligo gspE*Sac*Ifw, Oligo gspE*Eco*RIrv) end of the *gspE *gene. The amplified DNA fragments were digested by *SphI/SacI *and *EcoRI/SacI *and cloned into the pVS *Sph*I/*Eco*RI site to generate the pVS*gspE *vector. The resulting vector was mobilized by intergeneric conjugation [9] into *P. haloplanktis *TAC125, and the cells were plated at 4°C on TYP solid medium containing 30 μg/ml carbenicellin to select those clones in which a single recombination event occurred.

All PCR amplifications were performed in standard conditions [28]. The amplified fragments were cloned and their nucleotide sequences were checked to rule out the occurrence of any mutation during synthesis.

### Zymographic assay

*P. haloplanktis *TAC125 wild type and *gspE *mutant strains were grown in standard conditions and culture samples were collected at different growth phases as reported in the text. Samples were centrifuged at 10000 × *g *for 5 min at 4°C and the upper phase was collected for further analysis. The collected culture media were tenfold concentrated by Centricon (AMICON, exclusion size 5 kDa), and 12 μl were loaded onto a non reducing SDS-PAGE containing gelatine (1.5 mg ml^-1^). After electrophoresis, gel was soaked twice with 2.5% Triton X-100 (v/v) solution for a total of 60 min to remove SDS. The gel was then incubated in a developing buffer (50 mM Tris-HCl, pH 7.5, containing 5 mM CaCl_2_) for 12 or 18 (as indicated) hours at 15°C, rinsed with water, and stained with Coomassie blue R250. Areas of gelatine digestion, corresponding to proteolytic activities, were visualized as unstained regions in the gel.

### Protease inhibition assay

Tenfold concentrated culture supernatants of *P. haloplanktis *TAC125 wild type and *gspE*^- ^mutant cells were incubated with no inhibitors, or 10 mM EDTA, or 10 mM PMSF, or a combination of EDTA and PMSF (both at 10 mM final concentration) at 15°C for 20 hours. The samples were then subjected to protease zymographic assay.

## Authors' contributions

DDV and CC performed the experiments and helped to draft the manuscript. EP and MLT drafted the manuscript and designed and coordinated the study. All authors read and approved the manuscript

## References

[B1] Cornelis P (2000). Expressing genes in different Escherichia coli compartments. Curr Opin Biotechnol.

[B2] Makrides SC (1996). Strategies for achieving high-level expression of genes in *Escherichia coli*. Microbiol Rev.

[B3] Mergulhaõ F, Monteiro G, Cabral J, Taipa M (2004). Design of bacterial vector systems for the production of recombinant proteins in *Escherichia coli*. J Microb Biotechnol.

[B4] Mergulhaõ F, Monteiro G, Kelly A, Taipa M, Cabral J (2000). Recombinant human proinsulin: a new approach in gene assembly and protein expression. J Microb Biotechnol.

[B5] Liao YD, Jeng JC, Wang CF, Wang SC, Chang ST (2004). Removal of N-terminal methionine from recombinant proteins by engineered *E coli *methionine aminopeptidase. Protein Sci.

[B6] Cusano AM, Parrilli E, Marino G, Tutino ML (2006). A novel genetic system for recombinant protein secretion in the Antarctic *Pseudoalteromonas haloplanktis *TAC125. Microb Cell Fact.

[B7] Georgiou G, Valax P (1996). Expression of correctly folded proteins in *Escherichia coli*. Curr Opin Biotechnol.

[B8] Baneyx F (1999). Recombinant protein expression in *Escherichia coli*. Curr Opin Biotechnol.

[B9] Duilio A, Tutino ML, Marino G (2004). Recombinant protein production in Antarctic Gram-negative bacteria. Methods Mol Biol.

[B10] Vigentini I, Merico A, Tutino ML, Compagno C, Marino G (2006). Optimization of recombinant Human Nerve Growth Factor production in the psychrophilic *Pseudoalteromonas haloplanktis*. J Biotechnol.

[B11] Papa R, Rippa V, Sannia G, Marino G, Duilio A (2007). An effective cold inducible expression system developed in *Pseudoalteromonas haloplanktis *TAC125. J Biotechnol.

[B12] Parrilli E, Duilio A, Tutino ML, Margesin R, Schinner F, Marx JC, Gerday C (2008). Heterologous protein expression in psychrophilic hosts. Psychrophiles: from Biodiversity to Biotechnolgy.

[B13] Cusano AM, Parrilli E, Duilio A, Sannia G, Marino G, Tutino ML (2006). Secretion of psychrophilic alpha-amylase deletion mutants in *Pseudoalteromonas haloplanktis *TAC125. FEMS Microbiol Lett.

[B14] Feller G, Lonhienne C, Deroanne C, Libioulle J, Van Beeumen J, Gerday C (1992). Purification, characterization, and nucleotide sequence of the thermolabile alpha-amylase from the Antarctic psychrotroph *Alteromonas haloplanctis *A23. J Biol Chem.

[B15] Médigue C, Krin E, Pascal G, Barbe V, Bernsel A, Bertin PN, Cheung F, Cruveiller S, D'Amico S, Duilio A, Fang G, Feller G, Ho C, Mangenot S, Marino G, Nilsson J, Parrilli E, Rocha EPC, Rouy Z, Sekowska A, Tutino ML, Vallenet D, von Heijne G, Danchin A (2005). Coping with cold: the genome of the versatile marine Antarctica bacterium *Pseudoalteromonas haloplanktis *TAC125. Genome Research.

[B16] Johnson TL, Abendroth J, Hol WG, Sandkvist M (2006). Type II secretion: from structure to function. FEMS Microbiol Lett.

[B17] Yamagata A, Tainer JA (2007). Hexameric structures of the archaeal secretion ATPase GspE and implications for a universal secretion mechanism. EMBO J.

[B18] Chen Y, Shiue SJ, Huang CW, Chang JL, Chien YL, Hu NT, Chan NL (2005). Structure and function of the XpsE N-terminal domain, an essential component of the *Xanthomonas campestris *type II secretion system. Biol Chem.

[B19] Sandkvist M (2001). Biology of type II secretion. Mol Microbiol.

[B20] Sandkvist M (2001). Type II secretion and pathogenesis. Infect Immun.

[B21] Sandkvist M, Bagdasarian M, Howard SP, DiRita VJ (1995). Interaction between the autokinase EpsE and EpsL in the cytoplasmic membrane is required for extra-cellular secretion in *Vibrio cholerae*. EMBO J.

[B22] Blatny JM, Brautaset T, Winther-Larsen HC, Haugan K, Valla S (1997). Construction and use of a versatile set of broad-host-range cloning and expression vectors based on the RK2 replicon. Appl Environ Microbiol.

[B23] Kast P (1994). pKSS-a second-generation general purpose cloning vector for efficient positive selection of recombinant clones. Gene.

[B24] Ball G, Chapon-Hervé V, Bleves S, Michel G, Bally M (1999). Assembly of XcpR in the cytoplasmic membrane is required for extra-cellular protein secretion in *Pseudomonas aeruginosa*. J Bacteriol.

[B25] Turner LR, Lara JC, Nunn DN, Lory S (1993). Mutations in the consensus ATP-binding sites of XcpR and PilB eliminate extra-cellular protein secretion and pilus biogenesis in *Pseudomonas aeruginosa*. J Bacteriol.

[B26] Hanahan D (1983). Studies on transformation of *Escherichia coli *with plasmids. J Mol Biol.

[B27] Tascon RI, Rodriguez-Ferri EF, Gutierrez-Martin CB, Rodriguez-Barbosa I, Berche P, Va zquez-Boland JA (1993). Transposon mutagenesis in *Actinobacillus pleuropneumoniae *with a Tn10 derivative. J Bacteriol.

[B28] Sambrook J, Russell DW (2001). Molecular Cloning in A Laboratory Manual.

[B29] Feller G, D'Amico S, Benotmane AM, Joly F, Van Beeumen J, Gerday C (1998). Characterization of the C-terminal propeptide involved in bacterial wall spanning of alpha-amylase from the psychrophile *Alteromonas haloplanctis*. J Biol Chem.

